# Development of a new VMAT QA framework for Mobius3D using control-point specific EPID images

**DOI:** 10.3389/fonc.2024.1478118

**Published:** 2024-12-04

**Authors:** JaeHyun Seok, Hojin Kim, Min Cheol Han, Jihun Kim, Kwangwoo Park, Hyeonjeong Cho, Dohyeon Yoo, Jin Sung Kim

**Affiliations:** ^1^ Department of Integrative Medicine, Yonsei University College of Medicine, Seoul, Republic of Korea; ^2^ Medical Physics and Biomedical Engineering Lab (MPBEL), Yonsei University College of Medicine, Seoul, Republic of Korea; ^3^ Department of Radiation Oncology, Yonsei Cancer Center, Heavy Ion Therapy Research Institute, Yonsei University College of Medicine, Seoul, Republic of Korea; ^4^ Department of Radiation Oncology, Gangnam Severance Hospital, Yonsei University College of Medicine, Seoul, Republic of Korea; ^5^ Department of Radiation Oncology, Yongin Severance Hospital, Yonsei University College of Medicine, Yongin, Republic of Korea; ^6^ Department of Biomedical Sciences, Seoul National University College of Medicine, Seoul, Republic of Korea; ^7^ Oncosoft Inc., Seoul, Republic of Korea

**Keywords:** EPID, patient-specific QA, control-point specific QA, log-based QA, VMAT QA

## Abstract

**Purpose:**

This study presents novel quality assurance (QA) approach for volumetric modulated arc therapy (VMAT) that leverages frame-by-frame electronic portal imaging device (EPID) images integrated into Mobius3D for accurate three-dimensional dose calculations.

**Methods:**

Sequential EPID images for VMAT plans were acquired every 0.4-second by iView system and processed through iterative deconvolution to mitigate blurring from photon scattering. Deconvolved images were binarized to define multi-leaf collimator (MLC) positions. Pre-acquired box fluences determined optimal threshold for binarization and adjusted for detector shift depending on gantry and collimator angles. Sequential EPID images were re-scaled using pixel scaling factor (PSF) and converted to monitor unit (MU) proportional values. Generated EPID-based log file, including control-point specific MLC and monitor units (MU) information, were analyzed in Mobius3D for Gamma passing rate (GPR) of VMAT plans from 18 patients. Plan complexity indices were calculated and correlated with GPR.

**Results:**

Clinically appropriate threshold was defined to be 20000 that can extract accurate MLC data from the deconvolved binarized EPID images. Positional deviations due to gantry and collimator rotations were observed to be up to 4.5 pixels. Recalibrated EPID pixel values showed linearity with MU regardless of changes in dose rate. Consequently, average GPR for 18 patients evaluated using Mobius3D reached 95.2% ± 3.7%%, based on 3% dose difference and 3mm distance-to-agreement criterion. It was found that two plan complexity indices showed statistically significant correlation with GPR.

**Conclusion:**

This study successfully implemented novel measurement-based VMAT QA framework based on control-point specific EPID, based upon accurate MLC and MU data at each frame.

## 1 Introduction

Intensity-Modulated Radiation Therapy (IMRT) and volumetric modulated arc therapy (VMAT) represent advanced techniques in radiation therapy that allow for irradiate optimized dose to cancerous tissues while sparing the surrounding healthy tissue (1–4). The inherent complexity of these techniques occurs from their ability to modulate the intensity of the radiation beam and adjust the shape of the beam in real-time through the movement of multi-leaf collimator (MLC) (5, 6). Additionally, VMAT enhances this complexity by shifting the speed of the gantry rotation and the dose rate during the treatment, which necessitates sophisticated planning and execution strategies to achieve the desired therapeutic outcomes (7, 8). The precision of these therapies, while beneficial for patient care, underscores the critical need for comprehensive quality assurance (QA) protocols to ensure that the planned treatment is delivered accurately (9–11).

QA methods for treatment plans with intensity modulation, IMRT and VMAT, can be categorized into four techniques: measurement-based, detector-based, electronic portal imaging device (EPID)-based, and log-based methods. The measurement-based method employing film and ion chambers is the most trustworthy, while being labor-intensive with special care on phantom and ion chambers (12, 13). The detector-based method with 2D array diodes or ion chambers can provide more efficient measurement conditions, which still requires time and effort for detector setup (9, 14). In contrast, EPID-based method does not need additional measurement equipment, thus offering a quick and simple process. However, most commercialized systems acquire and analyze 2D composite fluences even for the arc-based treatment plans, disabling for detecting errors of dose deliverability in control point-specific fashion (15). The log-based method, relying on the recorded machine log files that include MLC and monitor units (MU) information at the time of acquisition, is known to be powerful and efficient. It can also provide independent 3D dose recalculation with actual equipment parameters (16, 17). Despite the conveniences, the indirect QA process may occasionally fail to detect MLC positional errors, which could lead to biased QA results (18).

This work presents a new VMAT QA method based on 2D sequential EPID images. The hybrid QA strategy is featured to integrate the advantages of both EPID and log-based QA approaches that potentially implements a fast and convenient measurement-based QA approach without compromising patient safety and treatment effectiveness. It begins with capturing control-point specific 2D sequential EPID images, which are used to precisely extract MLC positions and MU information during the dose delivery. Using this information, we generate a log file reflective of the MLC and MU details from the EPID images, which is then fed into a commercial QA software device to perform comprehensive IMRT QA analysis.

## 2 Methods

### 2.1 2D sequential EPID-based QA framework

**Figure 1:**
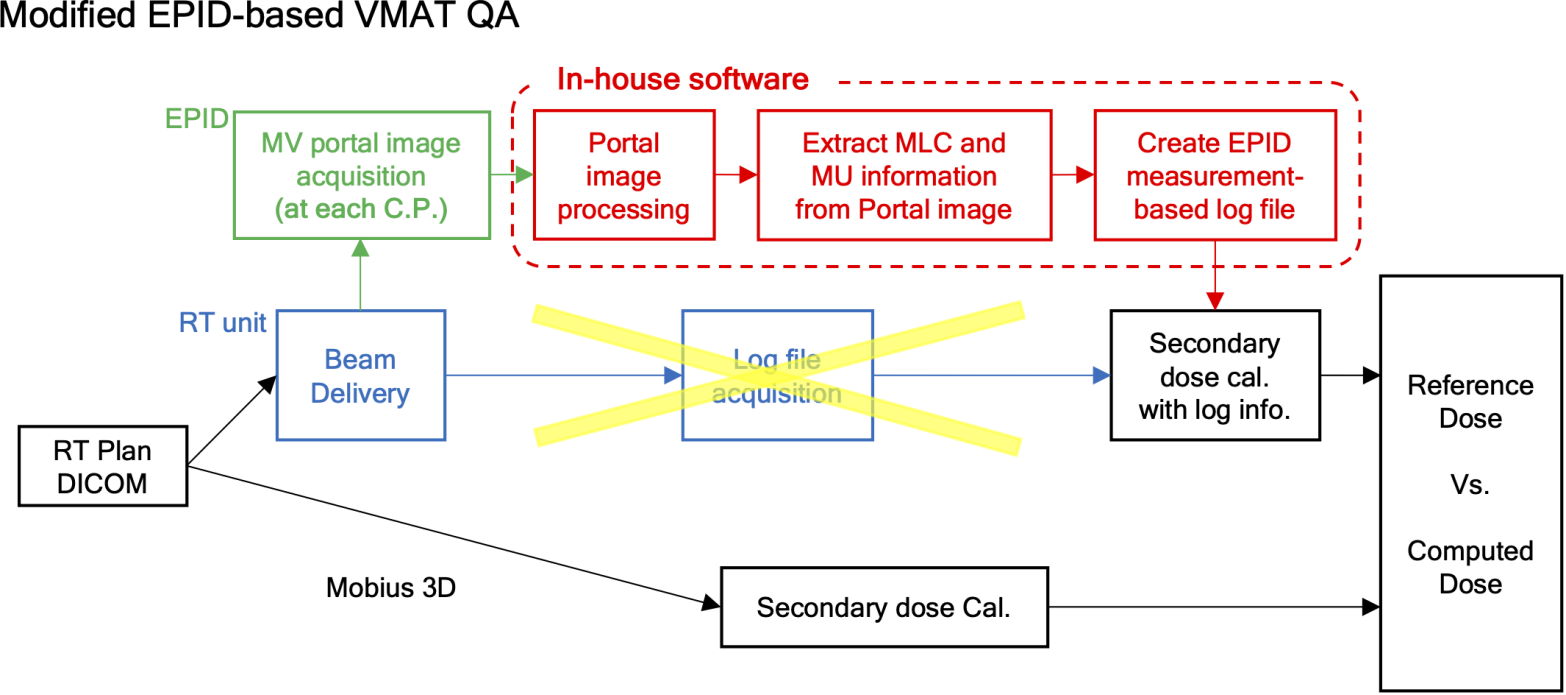
Framework of measurement-based control-point specific VMAT QA method based on EPID images.

**Figure 1** depicts the overall framework of our proposed sequential EPID images-based VMAT QA method, compared to the existing log-based QA method provided by Mobius 3D version 4.0.2 (Varian Medical System, Palo Alto, CA, USA). Basically, the previous approach performed the dose calculation for QA analysis with reference to the MLC and MU information saved in the machine log file following dose delivery. The proposed framework leveraged the sequential EPID images to obtain MLC and MU information during the dose delivery of VMAT rotational arc plans. The 2D EPID images acquired were processed by a de-convolution algorithm that can reduce the blurring effect caused by photon scattering. The de-blurred EPID images were then binarized to extract MLC positions at each time frame. The intensity of the images was adjusted by a specific indicator named pixel scaling factor (PSF) stored in the EPID log file, ensuring the proportionality to MU. The extracted MLC and MU information was fed into the Mobius3D software for comprehensive VMAT QA analysis. The details of each process were specified in the subsequent sections. All computational tasks, including the EPID image processing and the creation of the EPID-based log file, were performed using an in-house software in MATLAB version 9.13 (R2022b, Mathworks Inc., Natick, MA, USA).

### 2.2 MV EPID image acquisition

The execution of our proposed workflow necessitates the precise extraction of MLC positional information and MU at each 2D sequential EPID image. EPID images used for developing the proposed framework were acquired by the iView GT a-SI EPID equipped in Harmony (Elekta, Stockholm, Sweden). The iCom mode in iView GT generated the sequential 2D EPID images on a frame-by-frame basis at approximately 0.3-second time intervals with a resolution of 512 × 512 pixels in JPEG format. Also, it produced a single EPID log file, containing information of EPID acquisition time, total number of 2D sequential images, and PSF required for rescaling the EPID images. The number of spatially uniform control points designated in the RT plan DICOM files did not equal the number of temporally uniform control points in the EPID acquisition, in which the latter was greater than the former.

### 2.3 MV EPID image processing

Due to the original calibration performed in iView GT image acquisitions, the initial raw EPID images (*EPID_raw_*) were normalized to fit a 16-bit format whose values were inversely proportional to actual dose of radaition. To obtain an EPID image proportional to the dose (*EPID_processed_*), the raw pixel value of the *EPID_raw_* is subtracted from 65535 (2^16^ -1).


(1) $$EPID_{processed}=65535-\;EPID_{raw}$$

The *EPID_processed_* was blurred by various scattered photons and a distribution that does not accurately reflect the incident photon fluence, requiring additional image processing (19). To convert *EPID_processed_* to incident photon fluence, we employed a deconvolution algorithm with a Gaussian kernel, specifically through a 2D iterative deconvolution method (20). The blurred EPID image is mathematically represented as the convolution of true, incident photon fluence (*EPID_true_* in Equation 2) with a two-dimensional point-spread kernel (*K* in Equation 2).


(2) $$EPID_{processed}=\;EPID_{true}*K$$

The algorithm was performed by updating the blurred image (*EPID_approx(n)_*) from the true incident EPID image (*EPID_approx(0, n)_*), as seen in Equation 3. Then, the incident deblurred EPID image (*EPID_approx(n)_*) was updated with the newly defined the blurred image (*EPID_approx(n)_*), as expressed in Equation 4.


(3) $$EPID_{approx\left(n\right)}=\;EPID_{approx\left(0,n\right)}*K$$

(4) $$EPID_{approx\left(0,n+1\right)}=\;EPID_{approx\left(0,n\right)}+EPID_{processed}-\;EPID_{approx\left(n\right)}$$

It began iterating with an initial guess to be *EPID_approx(0,1)_ = EPID_processed_*, and stopped iterating until the difference between the updated blurred image (*EPID_approx(n)_*) and the given blurred image (*EPID_processed_*) became saturated. The finally updated 2D EPID image (*EPID_approx.(0,n+1)_*) was defined to be the deconvolved EPID image (*EPID_deconv_*) that closely represents the incident photon fluence.

### 2.4 Extraction of MLC and MU Information from EPID Images

**Figure 2:**
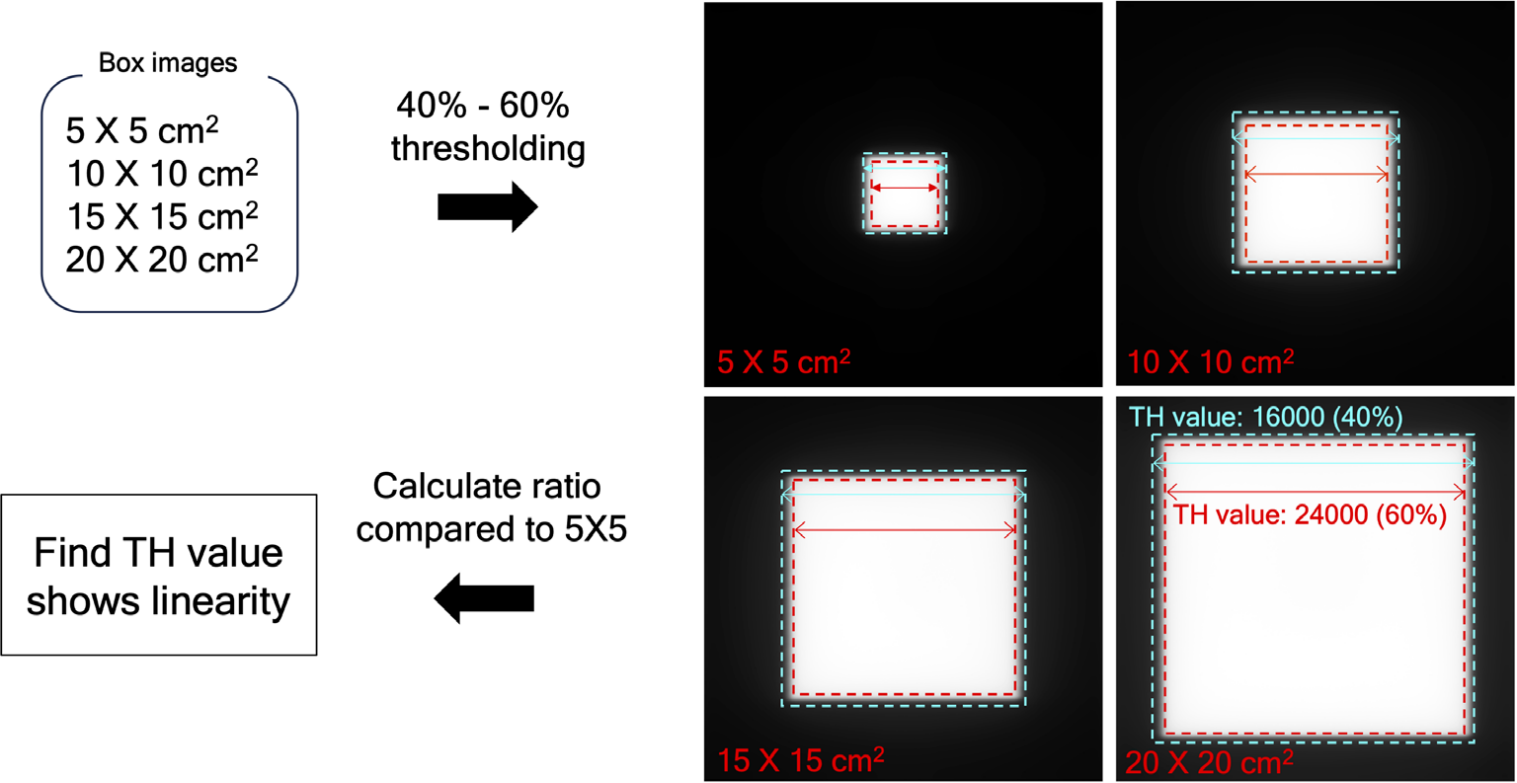
Determine the appropriate threshold value, using EPID image of box-shaped fluences with four dimensions by thresholding between 40% to 60% of the maximum pixel value. The range was found to maintain the quality of the binarized images consistently.

The de-convolved image (*EPID_deconv_*) needed to be binarized to extract MLC positional information, in which the appropriate threshold value needs to be considered. To find a clinically available value, we acquired *EPID_deconv_* of box-shaped fluences with different dimensions: 5 × 5, 10 × 10, 15 × 15, and 20 × 20 cm^2^. The four *EPID_deconv_* images with different field sizes were binarized using values from 40% to 60% of the maximum pixel value of the EPID images, in which the range of the thresholding operation was empirically determined. As specified in **Figure 2**, the optimal threshold was chosen, such that the number of pixels in the cross-plane direction of *EPID_deconv_* for 10 × 10 cm², 15 × 15 cm², and 20 × 20 cm² can hold linearity of 2 times, 3 times, and 4 times, respectively, compared to the number of pixels in the cross-plane direction of *EPID_deconv_* for 5 × 5 cm².

**Figure 3:**
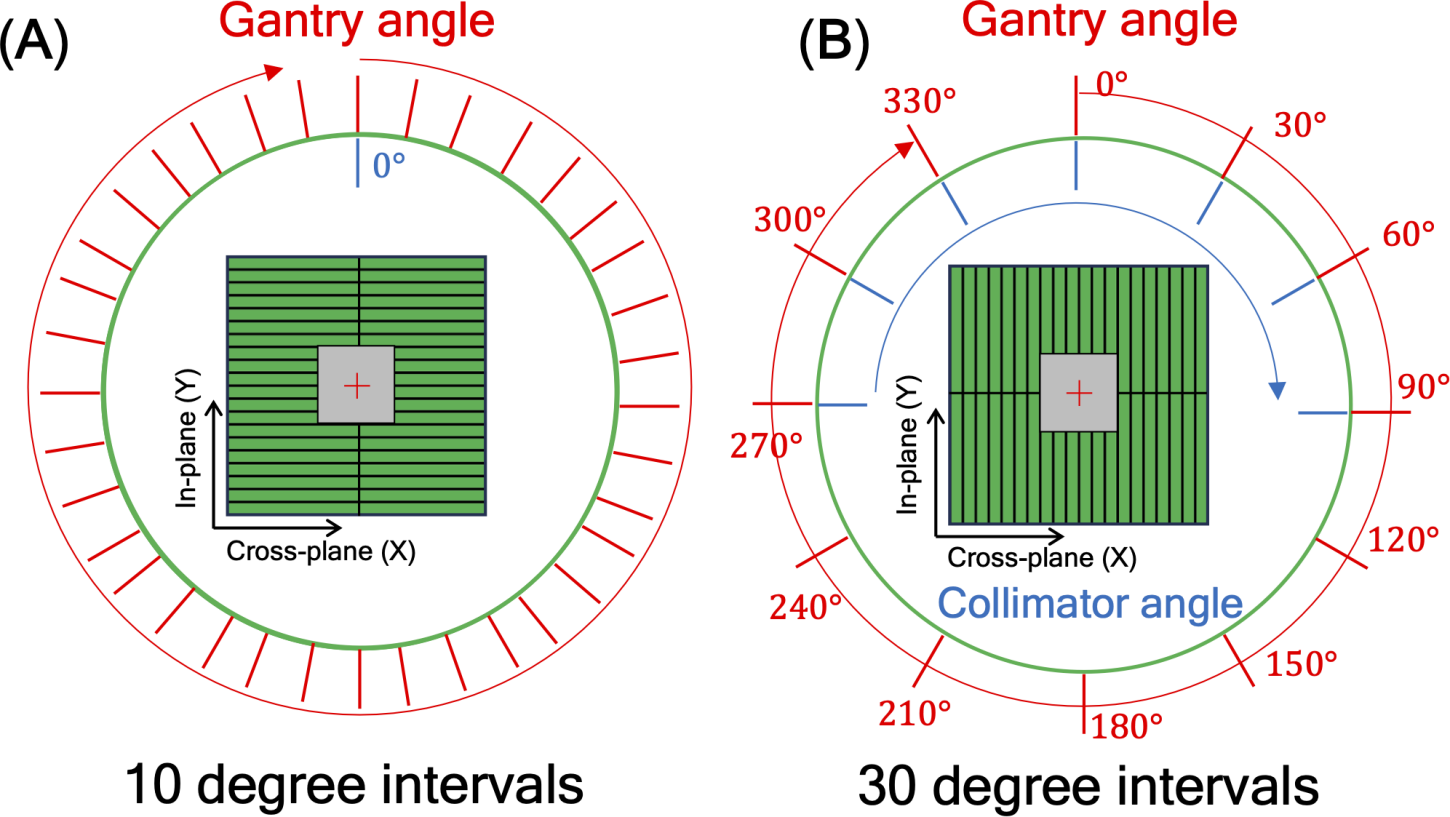
illustration of 10×10 cm^2^ box fluence used to calculate imaging center offset values to address **(A)** EPID detector sagging during the gantry angle rotation, and **(B)** additional center shift of EPID detector from collimator and gantry angle rotation. For offset value of EPID detector sagging, the gantry angles varied from 0 to 350 degrees with 10-degree intervals. For offset value of additional center shift, the gantry angles were adjusted in 30-degree intervals from 0 to 330 degrees, and for each gantry angle, the collimator angles were adjusted from -90 to 90 degrees in 30-degree intervals.

Another issue to be considered was a mechanical EPID detector shift from gantry and collimator rotations in VMAT treatment, potentially propagating errors in MLC positional information. To maintain consistent accuracy in identifying MLC positional information, a variety of EPID images were obtained with a standard 10 × 10 cm^2^ field dimension, while changing the gantry and collimator rotating angles. We have divided the sources of the imaging center offset values into two factors: 1) EPID detector sagging during the gantry angle rotation and 2) additional center shift from collimator rotation. EPID detector sagging from gantry rotation is unavoidable, inherent element that can affect the EPID image center shift, being independent of the collimator rotation. As illustrated in **Figure 3A**, the gantry angles varied from 0 to 350 degrees with 10-degree intervals to account for this. To additionally capture the secondary factor being affected by the collimator rotation, the EPID images were acquired at different collimator angles from -90 to 90 degrees in 30-degree intervals for each gantry angle, while the gantry angle changes from 0 to 330 degrees with 30-degree intervals, as illustrated in **Figure 3B**.

The intensity of actual dose, represented by MU, needed to be specified at each 2D sequential EPID images. The raw EPID images were re-scaled by Equation 1 to keep the proportionality to the dose of radiation. The actual intensity could be converted by referring to aforementioned frame-specific value, PSF (21). Equation 5 shows the conversion of the EPID image intensity to the effective global MU equivalent value (*EPID_MU_*).


(5) $$EPID_{MU}=EPID_{processed}\;/\;PSF$$

To assess the reliability of MU obtained via *EPID_MU_*, we conducted a MU linearity check. Specifically, it was assessed by ensuring the linearity of the MU equivalent values of two different sets of EPID images at 10 × 10cm^2^ field dimension, while changing the different MUs (1, 2, 3, 5, 10, 20, and 100 MUs) and different dose rates of 100, 300, and 500 MU/min. in delivering 100 MU.

### 2.5 Evaluation

The datasets used for this retrospective study were approved by the institutional review board of the Yonsei University Severance Hospital, Korea (4-2021-0869), which waived the need for informed patient consent to use patient-specific information of RT images, structures, dose and plan. To verify that the proposed QA method was properly implemented, we acquired sequential EPID images from eighteen VMAT plans using a 6 MV and 6 MV flattening filter free (FFF) beam energies with various body sites, as shown in **Table 1**. For each case, the MLC positional information and MU values extracted from the sequential EPID images was capsulized into a dynalog file-type format that is compatible to the Mobius 3D QA software. This allowed for conducting comprehensive VMAT QA analyses provided by the software, including 3D dose calculation and quantification of gamma passing rate (GPR). The patient-specific VMAT QA results were assessed by GPR at 3%/3 mm criterion for the 18 cases.

**Table 1:** Treatment sites, Beams, MUs of VMAT plans used to acquiring EPID frame-by-frame images.

Site	Beam	Fx. Dose	MU	MU/Fx. Dose
Bone	6	500	1639	3.28
Brain	6	200	734	3.67
Breast	6	320	923	2.88
Breast	6	520	1366	2.63
Breast	6	520	1816	3.49
Breast	6	520	1840	3.54
Breast	6	520	1865	3.59
Lung	6	300	558	1.86
Lung	6	400	769	1.92
Pancreas	6	600	1889	3.15
Pelvic seed	6	350	1971	5.63
PNS	6	200	582	2.91
Liver	6 FFF	700	2086	2.98
Lung	6 FFF	375	2281	6.08
Lung	6 FFF	500	1198	2.4
Lung	6 FFF	500	2078	4.16
Lung	6 FFF	700	1855	2.65
Ovary	6 FFF	350	871	2.49

To investigate the correlation between the complexity of the given VMAT plans and the deliverability of the VMAT plans (quantified by GPR), we calculated several plan complexity metrics using the open-source algorithm, available on GitHub (22) (http://github.com/AurelienCD/DeepHybridLearning_RadiotherapyQA_Depository_ManuscriptID_Diagnostics), which has been employed for previous studies (23–25). This work computed six plan complexity indices as follows: small aperture score (SAS), leaf travel (LT), modulation complexity score for VMAT plans (MCSv), combination of LT and MCSv (LTMCS), aperture area variability (AAV), and leaf sequence variability (LSV). SAS measures the proportion of small fields within a treatment plan. LT quantifies the total distance that MLC leaves travel during the delivery of a radiation therapy plan. MCSv evaluates the degree of modulation in the treatment, considering factors like variation in dose rate, gantry speed, and MLC positions across the arc of treatment. LTMCS combines the metrics of LT and MCSv to reflects both geometric changes in MLC positioning and the modulation of dose delivery. AAV measures the variability in the size of the apertures throughout the treatment. LSV evaluates the changes in leaf sequences and configuration during treatment. The plan complexity is interpreted to be incremental with larger SAS value and lower values of LT, MCSv, LTMCS, AAV, and LSV. Additionally, from the plan DICOM file, the MU and MLC information was employed for analyzing the plan complexity. We computed averaged mean and maximum MLC traveling distance (‘Mean_mean_MLC’ and ‘Mean_max_MLC’) between two consecutive control points of VMAT plans including standard deviations of those (‘STD_mean_MLC’ and ‘STD_max_MLC’). The average MU difference (Mean_MU) between two consecutive control points and its standard deviation (STD_MU) were also extracted. The correlation between twelve complexity indices and the gamma passing rate of each plan was assessed using Spearman’s rank correlation test. Spearman’s rank correlation test was performed in R version 4.3.3 (R foundation for Statistical Computing, Vienna, Austria).

## 3 Result

### 3.1 MLC and MU information extraction from sequential EPID images

**Figure 4:**
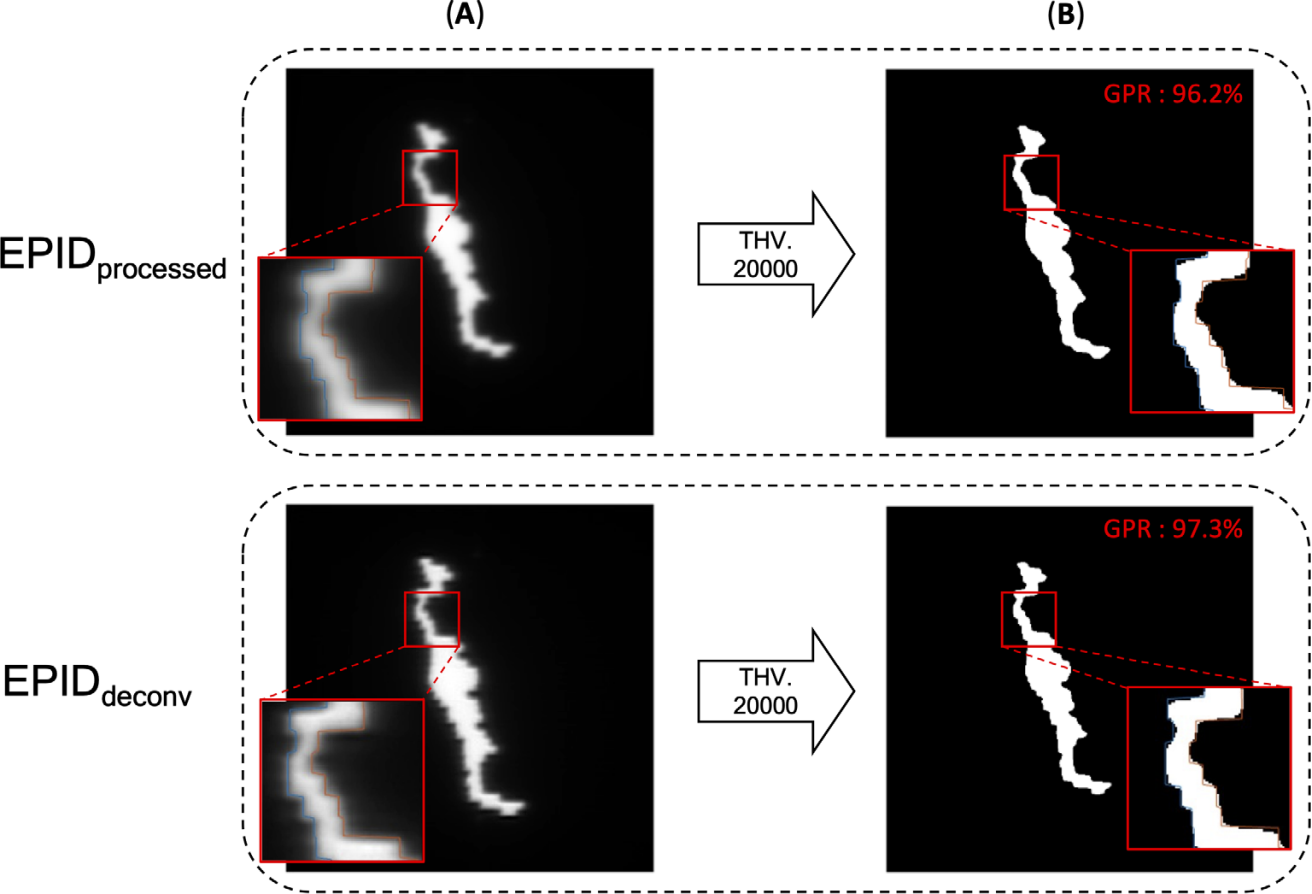
EPID images of before deconvolution (EPID_processed_) and after deconvolution (EPID_deconv_) is performed. **(A)** EPID image before binarization, **(B)** binarized EPID images by adequate threshold value. In the enlarged part, one can see that the boundaries of the binarized image and the MLC position do not align as well as after the deconvolution is applied. Additionally, GPR calculated by Mobius3D shows better result when the deconvolution is performed.

**Figure 5:**
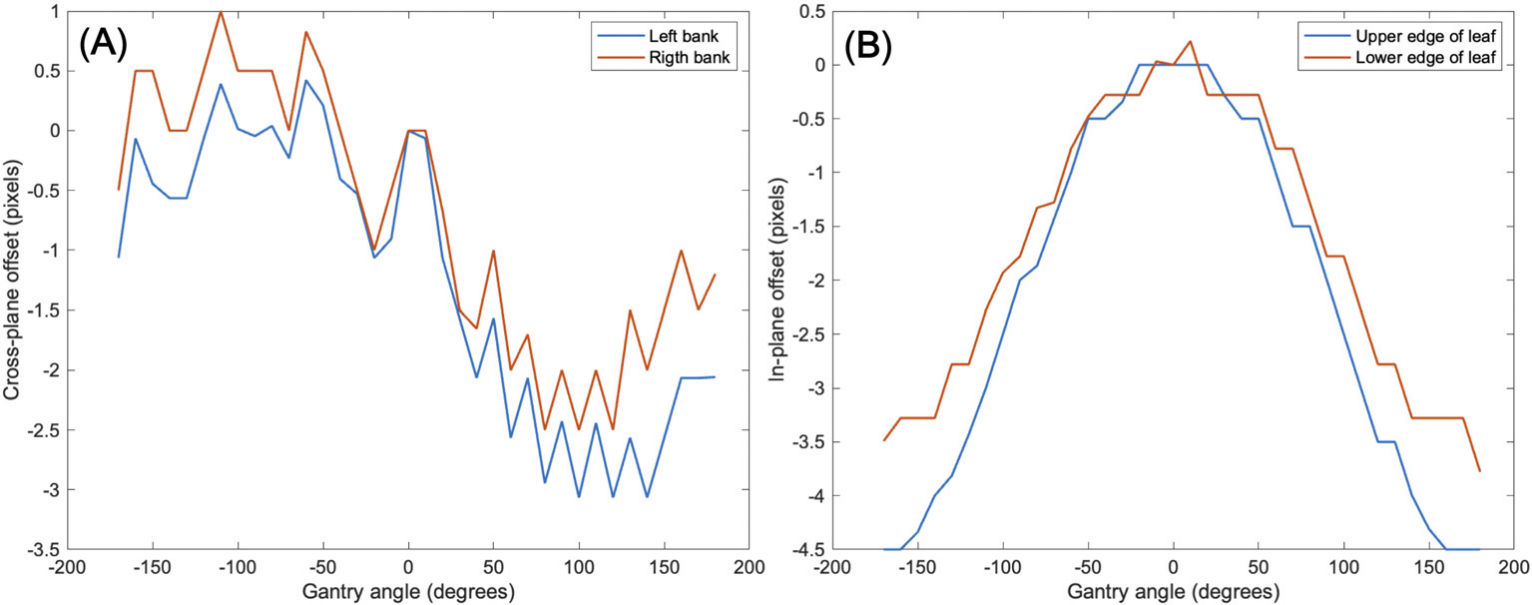
Imaging center offset values to address EPID detector sagging during the gantry angle rotation in the **(A)** cross-plane direction, **(B)** in-plane direction.

**Figure 6:**
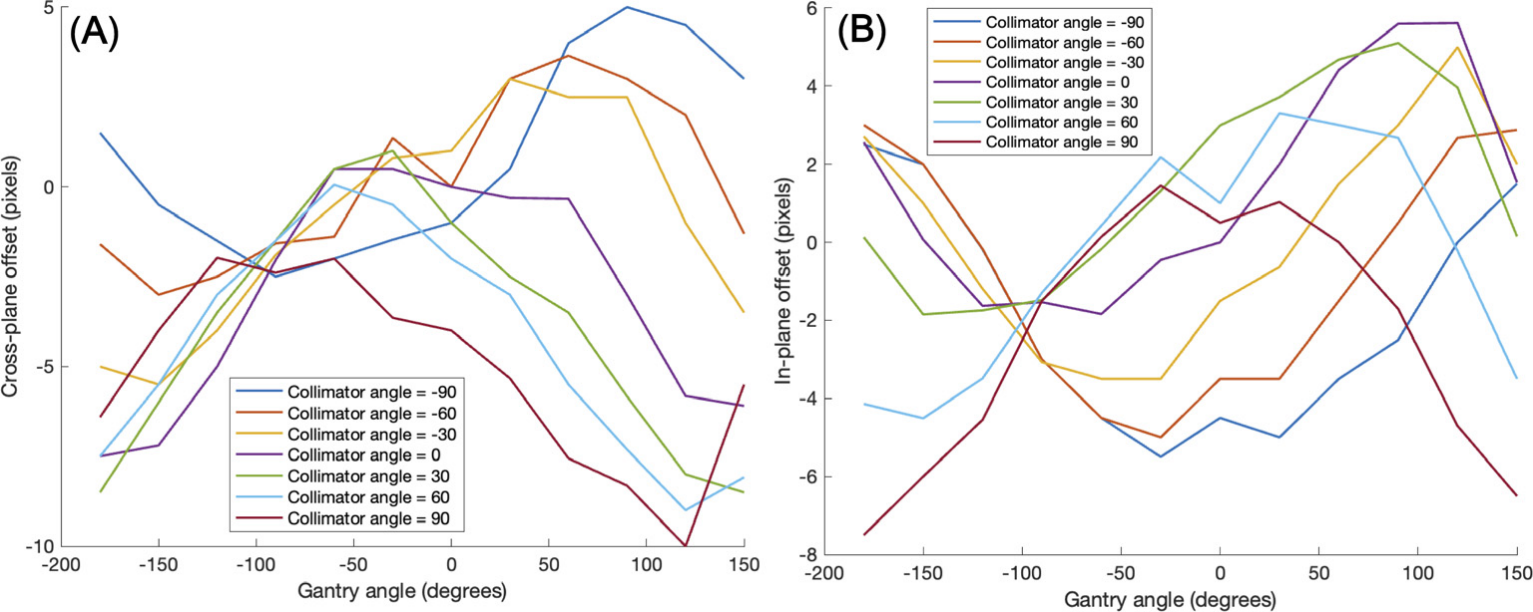
Imaging center offset values to address additional center shift of EPID detector caused by rotation of the gantry angle and collimator angle in the **(A)** cross-plane direction, **(B)** in-plane direction.

**Figure 7:**
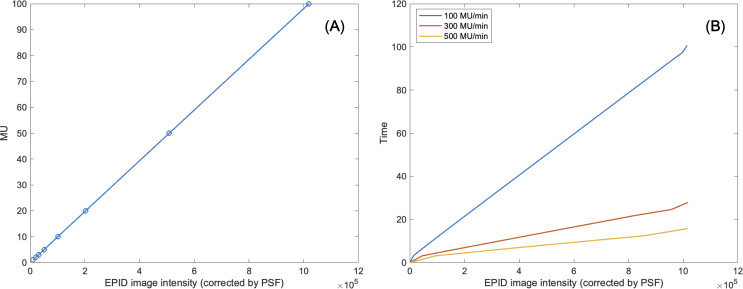
**(A)** Linearity of MU to EPID image intensity corrected by pixel scaling factor. **(B)** Consistency of EPID image intensity corrected by pixel scaling factor regardless of changes in dose rate.

**Table 2** shows linearity of the pixel counts of cross-plane of *EPID_deconv_* of box-shaped fluence with dimension of 5 × 5 cm^2^, 10 × 10 cm^2^, 15 × 15 cm^2^, 20 × 20 cm^2^. This analysis revealed optimal linearity within the threshold range of 19800 to 20200. Consequently, we selected the midpoint, 20000, as the threshold value, facilitating accurate MLC positional extraction from the EPID images. **Figure 4** shows the effect of deconvolution applied to *EPID_processed_*. The *EPID_deconv_* shows sharper edges than before it was applied, and the effect is more noticeable after the threshold is taken. Additionally, GPR calculated by Mobius3D shows better result when the deconvolution is performed. The imaging center offset values are calculated. The EPID detector sagging during the gantry angle rotation induces an offset ranging from -3.0654 to 0.4218 pixels on the left bank and from -2.5 to 1 pixel on the right bank in the cross-plane direction, as shown in **Figure 5A**. Additionally, an offset between -4.5 to 0 pixels is observed at the upper edge of the leaf in the in-plane direction, with an offset of -3.7792 to 0.2208 pixels at the lower edge of the leaf, as shown in **Figure 5B**. Regarding the shift in center of the EPID images caused by rotation of the collimator angles for the respective gantry angles, an offset is generated ranging from -10 pixels to 5 pixels in the cross-plane direction and from -7.5 pixels to 5.5 pixels in the in-plane direction, as shown in **Figure 6**. **Figure 7A** shows that the pixel values converted to *EPID_MU_* held linearity to the MUs delivered across 1, 2, 4, 10, 20, 50, and 100 MUs. Additionally, regardless of changes in dose rate, the pixel values of *EPID_MU_* remain consistent at 100 MU as shown in **Figure 7B**.

**Table 2:** Linearity of the pixel counts of cross-plan of deconvolved EPID image of box-shaped fluence with dimension of 5×5 cm^2^, 10×10 cm^2^, 15×15 cm^2^, 20×20 cm^2^.

Threshold value		16000	18000	19800	19900	20000	20100	20200	2000	24000
Linearity	10×10/5×5	1.973	1.985	2.000	2.000	2.000	2.000	2.000	2.010	2.028
	15×15/5×5	2.951	2.977	3.000	3.000	3.000	3.000	3.000	3.020	3.054
	20×20/5×5	3.928	3.964	4.005	4.005	4.005	4.005	4.005	4.031	4.083

### 3.2 GPR and plan complexity

The VMAT QA method based on 2D sequential EPID images was successfully executed by generating the EPID-based log file compatible to Mobius3D system for the 18 patient cases. For each plan, metrics such as GPR, DVH, and target coverage were calculated by Mobius3D. On average, the GPR remarked 95.2% ± 3.7%, in which most of the cases exceeded 90% in GPR, as shown in **Table 3**. However, the GPRs of two plans for lung and breast VMAT cases with 6 MV and 6MV FFF energy were below 90%, which might need to be reviewed before actual treatment. The MUs relative to the fractional dose were found to be high among the tested datasets, while some other cases with larger ratio of MUs to fractional dose resulted in the GPRs larger than 95%. This triggered on the necessity of quantifying and analyzing the plan complexity metrics.

**Table 3:** Gamma passing rate calculated from Mobius3D and six plan complexity indices of 18 VMAT plans.

Site	Beam	MU	GPR_prop_	GPR_mach_	SAS	MCSv	LT	LTMCS	AAV	LSV	MLC_mn_	MLC_Sn_	MLC_mx_	MLC_Sx_	MU_mean_	MU_STD_
Bone	6	1639	95.2%	98.7%	2.837	0.021	56.483	0.015	0.030	0.700	4.845	5.405	17.681	9.741	9.269	36.021
Brain	6	734	95.3%	99.2%	1.520	0.018	101.350	0.009	0.022	0.824	4.830	1.568	12.408	0.928	1.420	0.946
Breast	6	923	95.2%	96.1%	1.513	0.015	52.295	0.011	0.018	0.839	4.677	2.328	13.022	6.324	4.690	22.721
Breast	6	1366	93.9%	94.5%	1.583	0.016	53.823	0.012	0.018	0.859	5.025	3.029	13.008	7.544	12.074	90.754
Breast	6	1816	91.3%	88.3%	1.571	0.012	46.817	0.009	0.014	0.829	4.383	2.337	13.033	7.246	9.652	57.890
Breast	6	1840	88.9%	93.3%	1.614	0.013	61.378	0.009	0.016	0.836	6.377	2.250	12.359	1.139	7.694	8.004
Breast	6	1865	93.2%	93.5%	1.591	0.013	54.856	0.010	0.016	0.863	4.866	2.521	13.063	6.843	9.923	58.840
Lung	6	558	99.4%	99.9%	2.220	0.029	34.979	0.024	0.037	0.783	2.329	2.535	15.111	8.237	1.603	1.336
Lung	6	769	99.0%	99.4%	2.007	0.042	37.011	0.035	0.056	0.756	6.015	3.287	32.091	10.532	5.849	4.719
Pancreas	6	1889	99.2%	99.9%	2.173	0.021	68.965	0.014	0.029	0.731	4.051	2.851	17.876	6.005	5.309	3.375
Pelvic seed	6	1971	97.3%	98.6%	1.976	0.010	117.596	0.004	0.014	0.773	8.031	6.759	25.721	15.873	7.077	43.733
PNS	6	582	93.0%	97.9%	1.699	0.024	27.433	0.021	0.030	0.802	6.359	4.066	18.509	13.487	13.313	98.461
Liver	6 FFF	2086	93.8%	99.4%	1.709	0.020	80.769	0.012	0.023	0.846	4.143	2.179	20.620	2.141	5.853	7.093
Lung	6 FFF	2281	97.6%	99.8%	1.629	0.013	73.848	0.008	0.016	0.822	5.522	4.537	24.517	9.990	6.079	7.946
Lung	6 FFF	1198	98.6%	99.9%	2.819	0.041	41.279	0.033	0.057	0.720	2.568	3.077	13.909	7.294	6.537	10.363
Lung	6 FFF	2078	86.3%	99.6%	1.585	0.018	69.551	0.012	0.021	0.843	8.269	4.695	24.778	13.278	20.507	55.211
Lung	6 FFF	1855	99.5%	100.0%	2.473	0.032	54.840	0.023	0.045	0.710	3.699	3.888	16.581	6.126	5.213	6.570
Ovary	6 FFF	871	97.1%	99.2%	3.221	0.014	52.275	0.010	0.021	0.660	4.262	4.586	9.930	6.692	2.479	2.438
Spearman’s rank correlation test		Coefficient		0.737	0.588	0.491	-0.125	0.395	0.501	-0.660	-0.557	0.184	0.197	-0.010	-0.694	-0.650
		p-value		0.000	0.491	0.039	0.621	0.105	0.034	0.003	0.016	0.465	0.433	0.968	0.001	0.003

Correlation and p-value of Spearman’s rank correlation test between gamma passing rate from proposed method (GPR_prop_) and parameters: gamma passing rate from machine log (GPR_mach_), SAS, LT, MCSv, LTMCS, AAV, LSV, Mean_mean_MLC (MLC_mn_), STD_mean_MLC (MLC_Sn_), Mean_max_MLC (MLC_mx_), STD_max_MLC (MLC_Sx_), Mean_MU (MU_mean_), STD_MU (MU_STD_).

**Figure 8:**
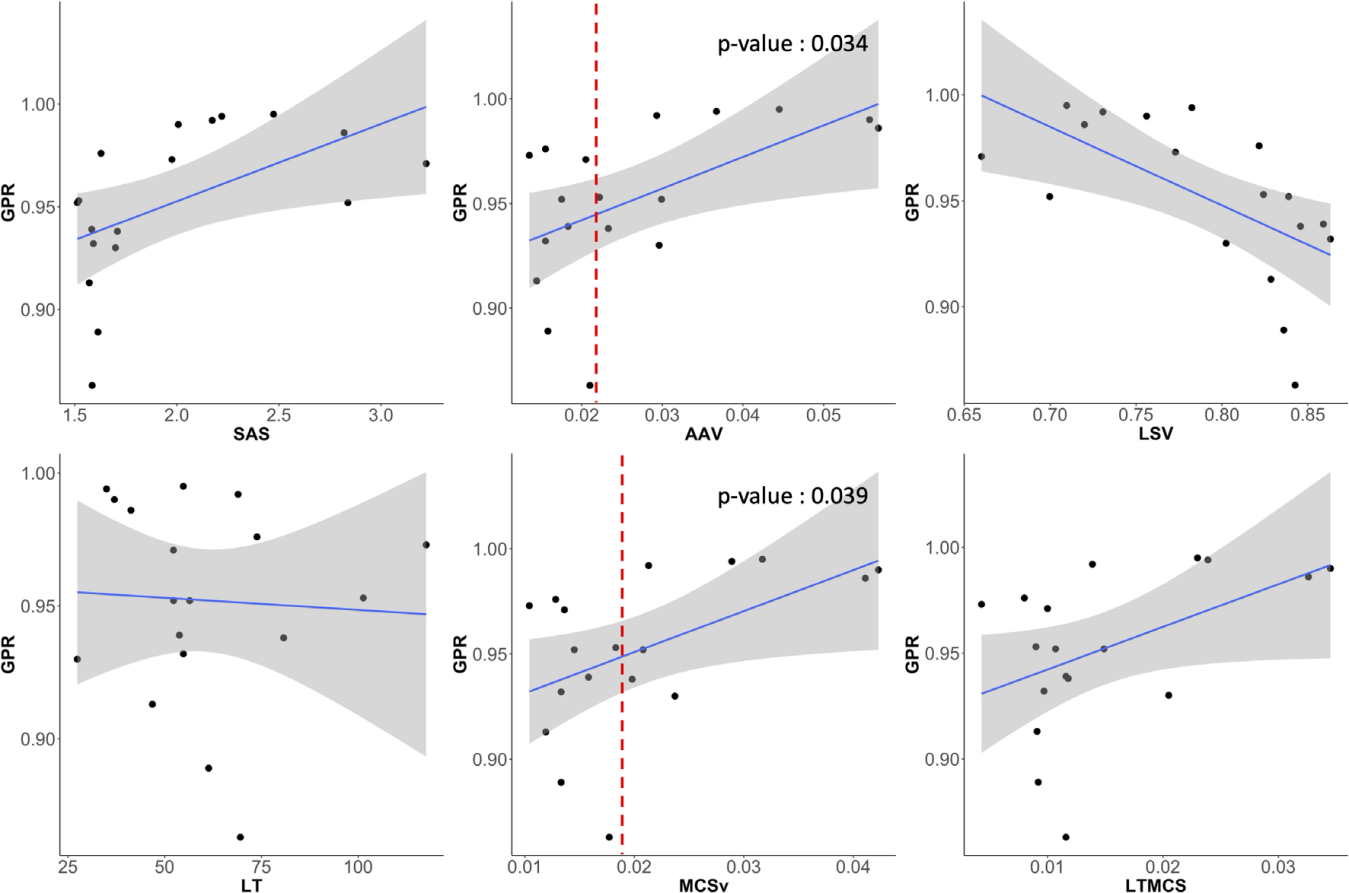
Scatter plots showing the correlation between GPR and six plan complexity indices (SAS, AAV, LSV, LT, MCSv, LTMCS). AAV and MCSv showed substantial correlations to the GPRs and had statistically significant influences.

The magnitude and pattern of the plan complexity values behaved different, which required for the correlation test with statistical analysis to ensure the relationship between GPRs and plan complexity indices. **Figure 8** shows scatter plots of correlation between six plan complexity indices and GPRs for the 18 VMAT plans, in which the solid lines represent Spearman’s rank correlation, and dashed lines mark threshold values for significant indices, suggesting critical levels of plan complexity. The correlations between three metrics (SAS, LSV, and LT) and GPRs were opposed to the expected, which were excluded in statistical analysis. For instance, the plan with greater value of SAS was supposed to yield low GPR, which behaved entirely opposite in our experiments. The remaining two of three plan complexity indices (AAV and MCSv) exhibited the large correlations around ±0.45 in appropriate direction, which led to the statistically significant influences (p < 0.05). Those complexity metrics, however, were not direct predictors for the GPRs as indicated by the dotted lines in red in **Figure 8**. The plans with similar complexity values to those with GPRs < 90% did not always result in the low GPRs.

**Figure 9:**
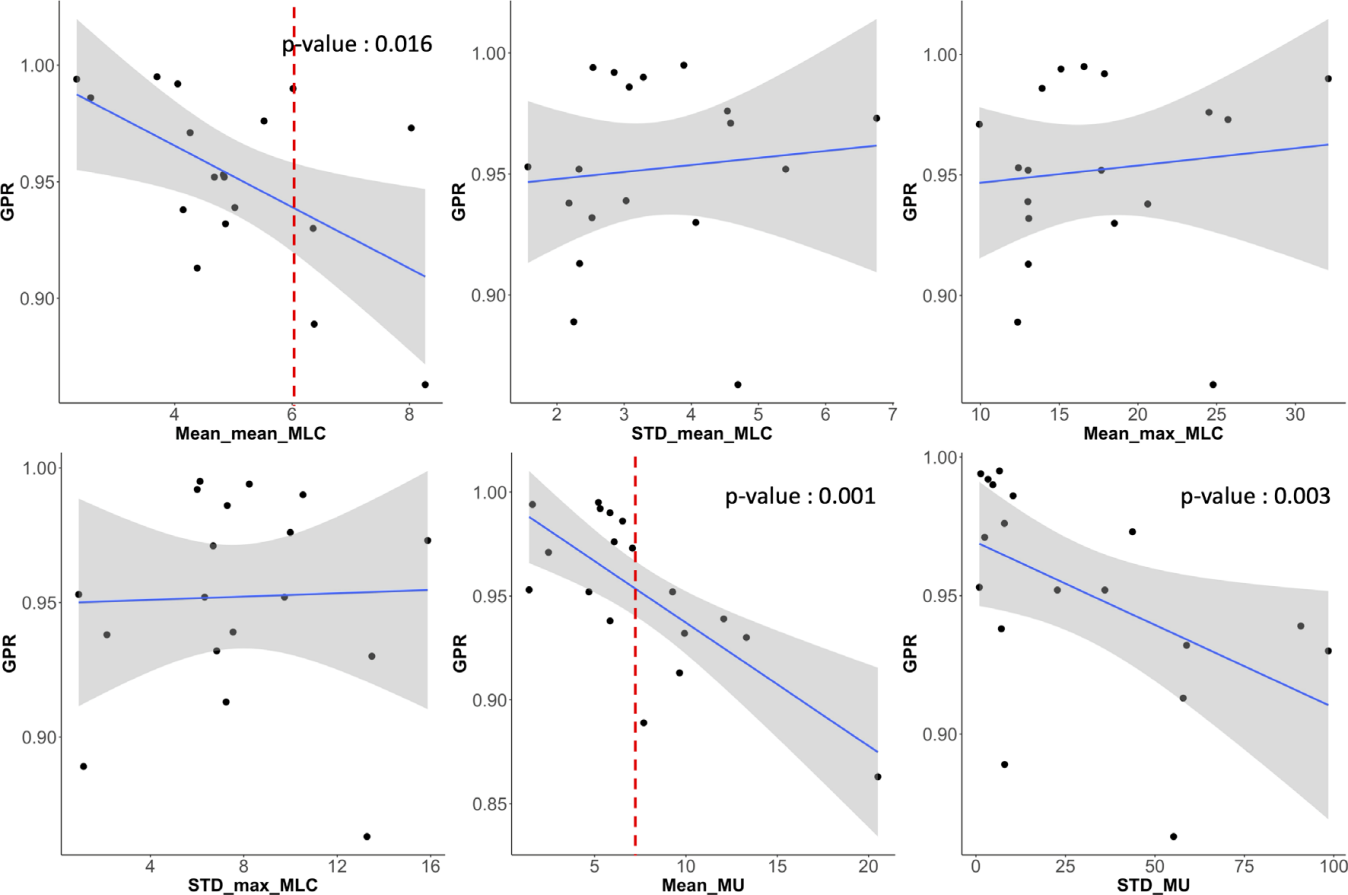
Scatter plots showing the correlation between GPR and six MLC and MU parameters (averaged mean and maximum MLC traveling distance (‘Mean_mean_MLC’ and ‘Mean_max_MLC’) and their standard deviations (‘STD_mean_MLC’ and ‘STD_max_MLC’), average MU difference (Mean_MU) between two consecutive control points and its standard deviation (STD_MU)). Averaged mean MLC traveling distance (Mean_mean_MLC) and averaged MU difference (Mean_MU) between two control points had also strong correlations to GPRs and had statistically significant influence.

**Figure 9** shows scatter plots of correlation between the direct MLC and MU-related complexity indices and GPRs. The mean of averaged MLC traveling distance (Mean_mean_MLC), averaged MU differences (Mean_MU), and standard deviation of MU differences (STD_MU) between two consecutive CPs revealed the reasonable direction of the correlations to the GPRs and statistically significant influences. Importantly, two indices (Mean_mean_MLC and Mean_MU) were rigorous predictors for the GPRs, in which the average MLC traveling distance of 6 cm and average MU difference of 8 MUs between two consecutive control points could offer a warning sign regarding plan deliverability.

We also compared the GPRs of EPID-generated (proposed) logs with those from machine-generated logs, yielding a significant correlation of 0.737 (p < 0.05). However, for VMAT plans using a 6MV FFF beam, GPR calculations using machine logs consistently showed results above 99%, indicating a limited capacity to differentiate plan complexity. Consequently, a Spearman’s rank correlation test for VMAT plans using 6MV beams only revealed a significant correlation coefficient of 0.919 (*p*= 0.00002).

## 4 Discussion

This study introduces a novel measurement-based control-point specific VMAT QA method that integrates the strengths of EPID-based QA and log-based QA. The primary goal was to utilize sequential EPID images captured during beam-on gantry rotation to mitigate occasional failure of detecting errors caused by machine fault due to lack of independent measurements in log-based QA. Furthermore, unlike traditional EPID-based QA that acquired and compared 2D composite fluences, our approach generated a dynalog file from MLC and MU information extracted from sequential EPID images, followed by facilitating independent 3D dose calculations. This enabled the acquisition of comprehensive information such as DVH and target dose, while maintaining the advantages of efficiency. Some previous works and the commercial products have attempted to develop patient-specific VMAT QA system with sequential EPID images. To our best knowledge, however, this study is the first trial of integrating both MLC and MU information with the measured frame-by-frame EPID images.

In technical aspects, the most challenging phase was to acquire the sequential EPID images. Differentiated from conventional EPID-based QA with only 2D composite fluence assigned to a whole or partial arc, frame-by-frame EPID images were required for generating a dynalog file in the new framework that is compatible to the commercial QA system, Mobius3D, for comprehensive QA analysis, including GPR based on 3D dose distributions. The Elekta linear accelerator allowed for an iCom connection mode that exported sequential 2D EPID images with a timing resolution of 0.3 seconds. The EPID images obtained from iCom mode, unfortunately, were blurred due to photon scattering in JPEG format, necessitating additional processing to extract accurate MLC and MU information. The blurred 2D sequential images were processed through iterative deconvolution algorithm and thresholding operator, which facilitated accurate and consistent extraction of placements of MLC leaves. Also, the PSF embedded in log files of sequential EPID images successfully converted the image intensities to MU-proportional values. The appropriateness of the threshold value in image binarization and PSF information for MU compatibility was ensured by independent linearity checks of the EPID images acquired with different MLC field sizes and different MU values.

The new QA framework developed in this work was successfully implemented, yielding an average GPR of 95.2% ± 3.7% across 18 VMAT plans. Most cases surpassed the clinically acceptable threshold of 90% GPR, while two out of the 18 plans exhibited GPRs below 90%. It led to the necessity of further investigation into the plan characteristics in relation to the GPRs. The 12 metrics were incorporated into the analysis, including six plan complexity metrics and six metrics based on direct MLC and MU transitional information from RT plan DICOM files. Two out of the six plan complexity metrics (AAV and MCSv) showed substantial correlations to the GPRs and had statistically significant influences. Two MLC and MU-directly associated metrics (averaged mean MLC traveling distance and averaged MU difference between two control points) had also strong correlations to GPRs and had statistically significant differences. Interestingly, those two MLC and MU-direct metrics implied strong potential for the GPR predictions, compared to the three plan complexity metrics. Plans with an average MLC traveling distance of 6 cm and an average MU difference of 8 MUs between two consecutive control points would be likely to leading to the GPRs < 90 with very few exceptions. In addition, for VMAT plans using 6MV FFF beams, despite variations in plan complexity indices, the machine log-based QA failed to detect the differences in GPR calculations, as observed in our study, thereby limiting its ability to screen non-deliverable plans during pre-treatment QA procedures. Contrarily, our EPID-generated logs successfully reflected the plan complexity of different plans in GPR calculations, ensuring a clinically secure methodology for patient-specific VMAT QA.

Despite the various strengths of our framework proposed in this work, there are a couple of limitations to be discussed. This study was conducted exclusively with a single linear accelerator, Harmony Pro by Elekta, which fortunately provided the 2D sequential EPID images. As stated previously, the presence of 2D sequential images was a key for the development of our proposed framework. Thus, it would be necessary to identify if a specific linear accelerator could allow us to obtain such sequential 2D EPID images for the applicability of this framework. Also, some critical parameters in determining a threshold value for image binarization and estimating mechanical offsets across gantry and collimator angles are likely to vary depending on the linear accelerators. It would have to be verified that such procedures proposed in this work are generalizable and applicable to the other linear accelerators. Additionally, the study was validated with only 18 plans. It possibly led to a bias in analyzing the results and identifying the factors associated with low GPRs. Therefore, further validation involving a wider range of equipment and a larger number of plans might be necessary to generalize the findings and improve the robustness of the proposed QA method.

## 5 Conclusion

This study successfully implemented a novel, measurement-based VMAT QA framework using control-point specific, frame-by-frame EPID images. The methodology involved extracting accurate MLC positions through iterative deconvolution and offset correction, as well as determining MU values using a PSF. The generated EPID-based log files were compatible with the Mobius3D system, facilitating accurate 3D dose calculations and comprehensive QA evaluations. Most of QA results met the clinical criteria, validating the effectiveness of the framework. In plans that did not meet the clinical criteria, we identified significant correlations between several plan complexity indices and the GPR. These findings highlight the framework’s potential to enhance VMAT QA processes. Future work will focus on validating this approach across a broader range of linear accelerators and treatment plans to ensure its generalizability and robustness.

## Data Availability

The datasets presented in this article are not readily available because they are confidential patient data and protected by institutional data use agreements. Requests to access the datasets should be directed to HK at HJHENRYKIM@yuhs.ac.
